# Novel PP2A-Activating Compounds in Neuroblastoma

**DOI:** 10.3390/cancers16223836

**Published:** 2024-11-15

**Authors:** Nazia Nazam, Laura V. Bownes, Janet R. Julson, Colin H. Quinn, Michael H. Erwin, Raoud Marayati, Hooper R. Markert, Sorina Shirley, Jerry E. Stewart, Karina J. Yoon, Jamie Aye, Michael Ohlmeyer, Elizabeth A. Beierle

**Affiliations:** 1 Division of Pediatric Surgery, Department of Surgery, University of Alabama at Birmingham, Birmingham, AL 35233, USA; nnazam@uabmc.edu (N.N.); lbownes@uabmc.edu (L.V.B.); jjulson@uabmc.edu (J.R.J.); chquinn@uab.edu (C.H.Q.); michael.erwin@northwestern.edu (M.H.E.); raoud.marayati@uabmc.edu (R.M.); hmarkert95@gmail.com (H.R.M.); 2025sns1@student.mtnbrook.k12.al.us (S.S.); jessy@uab.edu (J.E.S.); 2 Department of Pharmacology and Toxicology, University of Alabama at Birmingham, Birmingham, AL 35233, USA; kjyoon@uab.edu; 3 Division of Hematology/Oncology, Department of Pediatrics, University of Alabama at Birmingham, Birmingham, AL 35233, USA; jaye@uabmc.edu; 4 Atux Iskay LLC, Plainsboro, NJ 08536, USA; michael.ohlmeyer@gmail.com

**Keywords:** protein phosphatase 2A, neuroblastoma, ATUX-3364, ATUX-8385

## Abstract

Background: Neuroblastoma (NB) remains one of the deadliest pediatric solid tumors. Recent advancements aimed at improving outcomes have been insufficient, and patients with high-risk NB continue to have a poor prognosis. Protein phosphatase 2A (PP2A) is a tumor suppressor protein downregulated in many cancers, including NB. PP2A activation has been shown to affect the malignant phenotype in other solid tumors. The present studies aim to investigate the effects of two novel PP2A activators as a NB therapeutic. Methods: Four established NB cell lines and a patient-derived xenoline were utilized to study the effect on cell viability, proliferation, motility, and in vivo tumor growth using two novel tricyclic sulfonamide PP2A activators, ATUX-3364 and ATUX-8385. Results: ATUX-3364 and ATUX-8385 increased PP2A activity. These PP2A activators led to decreased viability, proliferation, and motility of NB cells. Treatment of animals bearing NB tumors with ATUX-3364 or ATUX-8385 resulted in decreased tumor growth in *MYCN*-amplified SK-N-BE(2) tumors. At the molecular level, PP2A-based reactivation led to dephosphorylation of MYCN-S62 and decreased MYCN protein expression. Conclusions: PP2A activators decreased NB cell viability, proliferation, and motility. In vivo experiments show that PP2A activators have more significant effects on tumorigenesis in *MYCN-amplified* tumors. Finally, phosphorylation of MYCN protein was decreased following treatment with novel sulfonamide PP2A activators. These data and mechanistic insights may be useful for developing new PP2A-based therapies that target MYCN for the treatment of NB.

## Simple Summary

Neuroblastoma is the third most common type of childhood cancer but is responsible for over 15% of all pediatric cancer deaths. Children with advanced disease have less than a 50% survival rate and are in desperate need of new treatment options. Protein phosphatase 2A (PP2A) is a protein that inhibits tumors, but it is turned off in neuroblastoma. We have previously shown that we can activate PP2A in neuroblastoma with certain drugs and decrease tumor growth, but these drugs also affect the function of the immune system. We now have new small molecules that activate PP2A without upsetting the immune system. We observed a decrease in tumor growth, proliferation, and motility with our novel PP2A activators. We feel that a better understanding of how activating PP2A inhibits neuroblastoma will lead to better treatments for these children.

## 1. Introduction

Children with high-risk neuroblastoma (NB) continue to have poor outcomes despite significant advances in the field of pediatric oncology [1]. Treatment for high-risk disease incorporates chemotherapy, surgical resection, stem cell transplant, radiation, immunotherapy, and maintenance with retinoic acid [2]. Despite this regimen, 50–60% of patients will suffer disease recurrence [3], making efforts to identify novel therapeutics imperative. *MYCN* gene amplification is the most important negative prognostic marker of NB [4]. Despite the strong link between *MYCN* amplification/over-expression and the transformed phenotype [5,6], the cellular factors that may be leveraged to alter the expression of the MYCN amplicon in NB cells remain unclear.

Protein phosphatase 2A (PP2A), a tumor suppressor, is downregulated in many malignancies [7,8], including NB [9,10], and the *MYCN* proto-oncogene is a well-characterized PP2A substrate [11]. PP2A reactivation has recently emerged as a medicinally tractable target for cancer therapeutics [12,13]. Strategies utilized to increase PP2A activation include compounds that inhibit the endogenous inhibitors of PP2A, namely I2PP2A (SET) and a cancerous inhibitor of phosphatase 2A (CIP2A), and small molecules that directly activate PP2A [10,12,13,14,15]. FTY720, a sphingosine analog that inhibits SET, decreased the malignant phenotype in several pediatric solid tumors, including NB [9], hepatoblastoma [14], and medulloblastoma [16]. Several small molecule activators of PP2A (SMAP), which emerged from a medicinal chemistry campaign to re-engineer tricyclic neuroleptics [17], have been shown to decrease tumor growth in models of lung [15], prostate [18], pancreatic [19], and breast [20] cancers and glioblastoma [21,22].

Our lab previously investigated tricyclic sulfonamide compounds that directly bind to the PP2A alpha scaffolding subunit, resulting in a conformational change and subsequent PP2A activation [10]. We showed that treatment with these compounds decreased the malignant phenotype of NB in vitro and in vivo [10]. Newer tricyclic sulfonamide compounds have been synthesized to provide improved water solubility, metabolic stability, and bioavailability. In this current study, we explore the therapeutic potential of two novel tricyclic sulfonamide PP2A activators in a diverse set of NB models to assess their effects on oncogenesis.

## 2. Materials and Methods

### 2.1. Cells and Cell Culture

This study employed four established human neuroblastoma cell lines and one human neuroblastoma patient-derived xenograft (PDX) cell line. The established cell lines include the SK-N-AS (*MYCN*-non-amplified cell line; AS, CRL-2137) and SK-N-BE(2) (*MYCN-amplified* cell line; BE, CRL-2271) obtained from the American Type Culture Collection (ATCC, Manasas, VA, USA). The other two cell lines are isogenic human NB cells SH-EP (*MYCN* non-amplified) and WAC2 (MYCN overexpressing), which were kindly gifted by M. Schwab (Deutsches Krebsforschungszentrum, Heidelberg, Germany) [22]. The SH-EP and WAC2 cell lines are unique in that they are isogenic for MYCN and have been extensively described [22]. All of the four established cell lines were maintained under standard culture conditions, 5% carbon dioxide, and 37 °C. SK-N-AS cells were maintained in Dulbecco’s modified Eagle’s medium (DMEM, Corning Inc., Corning, NY, USA) supplemented with 10% fetal bovine serum (FBS, Hyclone, Suwanee, GA, USA), 1 μg/mL antibiotic, penicillin/streptomycin (Sigma Aldrich, Burlington, MA, USA), 4 mM L-glutamine (Thermo Fisher Scientific Inc., Waltham, MA, USA), and 1 μM non-essential amino acid (Life Technologies, Grand Island, NY, USA). The SK-N-BE(2) cells were cultured in a 1:1 ratio of minimum Eagle medium (Corning) and Ham’s F-12 medium (Corning) with 10% FBS (Hyclone), 1 μg/mL penicillin/streptomycin (Sigma), 2 mM l-glutamine (Thermo Fisher Scientific), and 1 μM non-essential amino acid (Life Technologies). SH-EP and WAC2 cells were cultured in Roswell Park Memorial Institute (RPMI) 1640 medium (10-040-CV, Corning) with 10% FBS (Hyclone), 1 μg/mL penicillin/streptomycin (Sigma), and 2 mM L-glutamine (Thermo Fisher Scientific). All cell lines were tested for Mycoplasma infection (Universal Mycoplasma Detection Kit 30-1012K, ATCC) and were verified within the last year using short tandem repeat (STR) analysis (Genomics Core, University of Alabama at Birmingham (UAB), Birmingham, AL, USA).

A human NB PDX, COA6, was developed as previously described [23,24]. Briefly, after UAB Institutional Review Board (IRB) and UAB Institutional Animal Care and Use Committee (IACUC) approval (IRB 130627006 and IACUC-09803, respectively) and following parental informed consent and patient assent, a specimen was obtained from a pediatric patient with primary NB undergoing surgical excision. Fresh tissue was kept in serum-free RPMI 1640 medium, transported on ice to the laboratory, and implanted with 25% Matrigel (BD Biosciences, Franklin Lakes, NJ, USA) into the flank of an athymic nude mouse (Fredricks, Charles River, Wilmington, MA, USA). When tumors measured 2000 mm^3^, mice were euthanized and tumors harvested. A portion of each tumor was sequentially passed into another animal for PDX maintenance, while separate portions were dissociated (tumor dissociation kit, Miltenyi Biotec, San Diego, CA, USA), and single cells were used for experimentation. COA6 cells recapitulate the properties of the parent tumor after several passages [23]. COA6 PDX cells were monitored using histology and short tandem repeat analysis (Heflin Center for Genomic Sciences, UAB, Birmingham, AL, USA). Real-time PCR (qPCR) assessed the percentage of human and mouse DNA contained in the PDX cells to ensure no murine contamination (TRENDD RNA/DNA Isolation and TaqMan QPCR/Genotyping Core Facility, UAB, Birmingham, AL, USA). PDX cells do not propagate in culture. They were maintained in standard culture conditions for experimentation in neurobasal media (Life Technologies, Carlsbad, CA, USA) supplemented with B-27 supplement without Vitamin A (Life Technologies), N2 supplement (Life Technologies), amphotericin B (250 μg/mL), gentamicin (50 μg/mL), l-glutamine (2 mM), epidermal growth factor (10 ng/mL; Miltenyi Biotec), and fibroblast growth factor (10 ng/mL; Miltenyi Biotec).

### 2.2. Reagents and Antibodies

For Western blotting, the following primary antibodies were utilized: rabbit polyclonal anti-MYCN (9405S), anti-MYCN-S62 (13748S) from Cell Signaling (Danvers, MA, USA), rabbit polyclonal anti-CIP2A (ab99518), rabbit polyclonal anti-cleaved PARP (ab3565) from Abcam (Cambridge, MA, USA), rabbit polyclonal anti-I2PP2A (SET, 55201-AP) from Proteintech (Rosemont, IL, USA), rabbit monoclonal anti-vinculin (13901S), and mouse monoclonal beta-actin (A1978, Sigma). Mouse monoclonal anti-GAPDH (MAB374) was from Millipore Sigma (Billerica, MA, USA). PP2A-activating compounds ATUX-3364 and ATUX-8385 were provided by Atux Iskay, LLC (Plainsboro, NJ, USA).

### 2.3. Synthesis of ATUX-8385 and ATUX-3364

ATUX-8385 and ATUX-3364 were synthesized by Dr. M. Ohlmeyer using a modification of the route described in Ohlmeyer and Zaware in the published patent application US 2018-0251456. The compounds were stored in the dark in a sealed container at room temperature. As compared to the previous prototype compound, DBK-1154 [10], these new compounds are novel. The tricyclic in ATUX-8385 and ATUX-3364 is difluorocarbazole versus DBK-1154, where it is dibenzoazepine. Further, the central ring constraint in ATUX-8385 and ATUX-3364 is piperidine versus a cyclohexyl ring in DBK-1154 [10]. This piperidine ring is a basic moiety that provides options for the formulation of the compounds as salts.

### 2.4. Pharmacokinetic Studies ATUX-8385

Pharmacokinetic (PK) studies of ATUX-8385 were conducted by Eurofins. Briefly, ATUX-8385 was administered at 1 mg/kg intravenously (IV) or 30 mg/kg oral (PO) to male mice, as is the experimental choice of the Contract Research Organization. At euthanasia, samples were collected and stored at −70 °C until processing. The plasma samples were collected at 0.05, 0.167, 0.5, 1, 2, 4, 8, and 24 h(s) after IV treatment, and at 0.167, 0.5, 1, 2, 4, 6, 8, and 24 h(s) after PO treatment. The plasma samples were processed using acetonitrile precipitation and analyzed by liquid chromatography–tandem mass spectrometry (LC-MS/MS). Since ATUX-8385 and ATUX-3364 are enantiomers and preliminary in vitro evaluation did not suggest significant differences between the two compounds, PK analyses were completed only on ATUX-8385.

### 2.5. Immunoblotting

Post-treatment, cell lysis was completed in radioimmunoprecipitation (RIPA) buffer. Phosphatase inhibitors (P5726, Sigma), protease inhibitors (P8340, Sigma), and phenylmethanesulfonylfluoride (PMSF, P7626, Sigma) were added to make a complete RIPA buffer. Lysis was performed for 60 min at 4 °C followed by centrifugation at 17,000 rpm for 30 min (4 °C). The protein concentration was determined using the Pierce BCA Protein Assay (Thermo Fisher Scientific), and protein separation was completed on sodium dodecyl sulfate polyacrylamide (SDS-PAGE) gel-based electrophoresis. Targeted protein size was determined using molecular weight markers (Precision Plus Protein Kaleidoscope, Bio-Rad, Hercules, CA, USA). Antibodies were used according to the manufacturers’ suggestions. Immunoblots were developed with Luminata Classico or Crescendo Western horseradish peroxidase substrate (EMD Millipore, Burlington, MA, USA) on X-ray film. Anti-beta-actin, anti-vinculin, or anti-GAPDH served as internal loading controls.

### 2.6. Protein Phosphatase 2A (PP2A) Activation

SK-N-AS, SK-N-BE(2), SH-EP, or WAC2 cells (1 × 10^6^) were treated with ATUX-3364 or ATUX-8385 (SK-N-AS and SK-N-BE(2) 4 µM; SH-EP and WAC2 12 µM) for 24 h and lysed using HEPES ((4-(2-hydroxyethyl)-1-piperazineethanesulfonic acid, 10 mM) (AC215001000, Thermo Fisher Scientific), MgCl_2_ (1.5 mM) (MX0045-4, EM Science, Gibbstown, NJ, USA), KCl (10 mM) (02-003-741, Thermo Fisher Scientific), phenylmethylsulfonyl fluoride (0.5 mM) (PMSF, Sigma Aldrich), dithiothreitol (0.5 mM) (EC-601, National Diagnostics, Atlanta, GA, USA), and Igepal CA-630 (0.2%) (I7771, Sigma Aldrich) on ice for 20 min. Cell lysates were centrifuged at 17 000 rpm for 30 min at 4 °C. A PP2A Immunoprecipitation Phosphatase Assay Kit (17–313, EMD Millipore) was utilized per the manufacturer’s protocol to evaluate the activity of PP2A. Experiments were completed with at least three biological replicates, and data were reported as mean fold change ± standard error of the mean (SEM).

### 2.7. Cell Viability and Proliferation

Cell viability was determined by alamarBlue assay (Thermo Fisher Scientific) and proliferation was detected with CellTiter 96 Proliferation assay (Promega, Madison, WI, USA). NB cell lines (15,000 cells for viability or 5000 for proliferation) or COA6 PDX cells (30,000 for viability or 10,000 for proliferation) were seeded in 96-well cell culture plates followed by treatment with increasing doses of ATUX-3364 or ATUX-8385 for 24 h. Doses were as follows: established cell lines, 0–20 µM; PDX COA6 cells, 0–25 µM. Respective dyes (10 µL) were added, and absorbance measurement was completed with a microplate reader (Epoch Microplate Spectrophotometer, BioTek Instruments, Winooski, VT, USA). A wavelength of 570 nm was used for alamarBlue and 490 nm for CellTiter 96 with 600 nm serving as the reference for both. A minimum of three biological replicates for each experiment were completed, and data were reported as fold change ± SEM.

### 2.8. Cell Motility

Migration was investigated using a monolayer wound healing (scratch) assay. Established NB cells (5 × 10^4^) were plated in 12-well plates. Once they reached 80% confluence, a sterile 200 μL pipette tip was used to make a standard scratch in the cell layer, and they were treated with ATUX-3364 or ATUX-8385 (SK-N-AS and SK-N-BE(2) 4 μM; SH-EP and WAC2 12 μM); with doses based upon previously calculated values for a lethal dose of 50% (LD_50_). Photographs of the plates were obtained at 0, 12, 24, and 36 h. The ImageJ MRI Wound Healing Tool (https://imagej.net, accessed on 26 November 2021) quantified the open wound area as previously described [25]. In PDX cells, migration was assessed using modified Boyden chamber assays. Micropore inserts with 8 µM pores (Corning) were utilized in 24-well plates. The insert bottoms were coated with human laminin (10 µg/mL, 150 μL, EMD Millipore). COA6 PDX cells were treated for 24 h with ATUX-3364 or ATUX-8385 (6 μM), plated (4 × 10^4^ cells), allowed to migrate through the membrane for 72 h, and the insert membranes were fixed using 4% paraformaldehyde and stained with 1% crystal violet for 15 min. Inserts were photographed, and migration was quantified with ImageJ (https://imagej.net, accessed on 26 November 2021). Scratch assays were reported as the fold change area remaining open at 36 h, and modified Boyden chamber assays were reported as the mean number of migrated cells ± SEM. Experiments were completed with at least three biological replicates.

### 2.9. Animal Statement

The UAB Institutional Animal Care and Use Committee (IACUC-09064, IACUC-09803) approved all animal experiments, and the studies were conducted within institutional, national, and NIH guidelines.

#### In Vivo Tumor Growth

SK-N-AS or SK-N-BE(2) (1.5 × 10^6^) cells in 25% Matrigel (BD Biosciences) were injected into the right flank of 6-week-old female athymic nude mice (Fredricks) (n = 7 for SK-N-AS vehicle treated, n = 6 for SK-N-AS experimental groups, n = 5 for SK-N-BE(2) vehicle treated, n = 6 for SK-N-BE(2) ATUX-3364, and n = 7 ATUX-8385 treatment groups). Tumors were measured thrice per week with calipers, and tumor volumes were calculated by the formula (width^2^ × length)/2, where the width was the smaller measurement. When tumors reached a volume of 60–100 mm^3^, animals were randomized to three groups to receive 100 µL twice daily by oral gavage of vehicle [N,N-dimethylacetamide (DMA, 271012, Sigma Aldrich) and Kolliphor HS 15 (Solutol, 42996, Sigma Aldrich)], ATUX-3364 (50 mg/kg in DMA and Solutol), or ATUX-8385 (50 mg/kg in DMA and Solutol); dosing was based on previously published data using in vivo cancer models [10,23]. Animals were weighed daily and were humanely euthanized in their home cages with CO_2_ and cervical dislocation 14 (SK-N-AS) or 21 (SK-N-BE(2)) days after treatment initiation, when the tumor volume reached 2000 mm^3^, or when animals met IACUC parameters. Because COA6 cells grow tumors unpredictably and erratically in mice, these PDX cells were not utilized for in vivo studies, but all COA6 tumors were propagated in mice.

### 2.10. Statistical Analysis

All in vitro experiments were performed with at least three biological replicates, and data were reported as the mean ± SEM [26]. ANOVA or Student’s *t*-test was utilized as appropriate, and statistical significance was defined at *p* ≤ 0.05.

## 3. Results

### 3.1. Novel PP2A Activators

**Figure 1:**
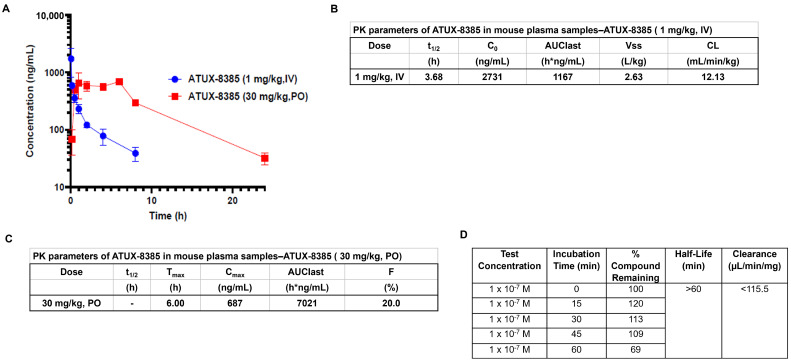
Pharmacokinetic studies for ATUX-8385. (**A**–**C**) Pharmacokinetic studies were completed by Eurofins. ATUX-8385 was administered at 1 mg/kg intravenously (IV) or 30 mg/kg oral (PO) to mice. The plasma samples were collected, processed using acetonitrile precipitation, and analyzed by LC-MS/MS. (**A**) Plasma concentrations were sustained longer with PO administration. (**B**,**C**) Pharmacokinetic data in tabular form for IV and PO dosing of ATUX-8385. (**D**) Pharmacokinetic data for in vitro metabolism for ATUX-8385. t_1/2_ = half-life; C_0_ = concentration at time zero; AUClast = area under the curve from time of administration up to the time of the last quantifiable concentration; Vss = steady-state volume of distribution; CL = clearance; T_max_ = time to maximum concentration; C_max_ = maximum concentration; F = bioavailability.

ATUX-8385 and ATUX-3364 are enantiomers, so PK data were obtained solely for ATUX-8385. The pharmacokinetic data for IV and PO administration are summarized in graphic (Figure 1A) and tabular form (Figure 1B,C, respectively). The in vitro metabolism results are presented in Figure 1D (tabular form). Plasma levels of 1–2 micromolar for approximately 8 h are observed after a bolus oral dose at 30 mg/kg in the PK experiment (Figure 1A,C). The in vitro microsome (oxidative) stability for ATUX-8385 is <115.5 µL/min/mg (Figure 1D) compared to DBK-1154, which was 234 µL/min/mg [10], making ATUX-8385 more stable by a factor of two. For the in vivo studies, the clearance of ATUX-8385 IV is 12.3 mL/min/kg (Figure 1B) compared to DBK-1154, which was 32.5 mL/min/kg [10], making ATUX-8385 more stable by a factor of nearly three times.

### 3.2. PP2A Activity Increases with ATUX-3364 and ATUX-8385 Treatment

**Figure 2:**
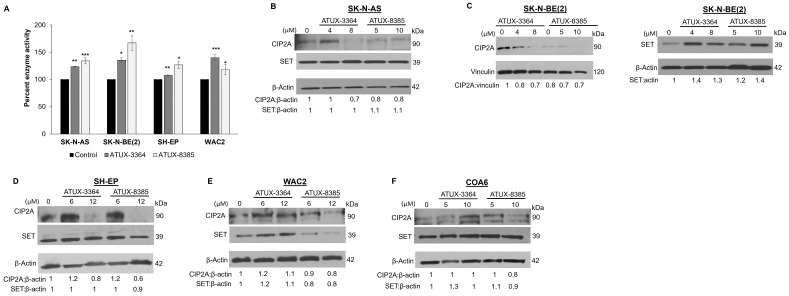
ATUX-3364 and ATUX-8385 affect PP2A enzyme activity. SK-N-AS, SK-N-BE(2), SH-EP, or WAC2 cells were treated with ATUX-3364 or ATUX-8385 for 24 h and PP2A activity measured. Percent PP2A enzyme activity was compared to that of untreated (control) cells. (**A**) PP2A activation was significantly increased in SK-N-AS with ATUX-3364 or ATUX-8385. Similarly, PP2A was significantly activated in the three other cell lines with both compounds. There was no significant difference in PP2A activation between the two compounds in any of the cell lines (**B**). In SK-N-AS cells, expression of CIP2A was decreased at higher doses and SET expression was unchanged following ATUX-3364 or ATUX-8385 treatment. (**C**) Expression of SET (**left panel**) was increased in SK-N-BE(2) cells and CIP2A decreased (**right panel**) after treatment. (**D**) Treatment with ATUX-3364 or ATUX-8385 led to decreased CIP2A expression but did not alter SET expression in SH-EP cells. (**E**) Treatment of WAC2 cells with ATUX-8385 decreased CIP2A and SET expression but ATUX-3364 increased these proteins at higher doses. (**F**) CIP2A expression was increased with the highest dose of ATUX-8385 but SET expression was variably affected in the NB PDX COA6 cells. Data reported as mean fold change ± standard error of the mean (SEM), and experiments were repeated with at least three biologic replicates. * *p* ≤ 0.05, ** *p* ≤ 0.01, *** *p* ≤ 0.001. The uncropped bolts are shown in Supplementary Materials.

We sought to determine the target activity following treatment with these new compounds in SK-N-AS, SK-N-BE(2), SH-EP, and WAC2 cells. Cells were treated with ATUX-3364 or ATUX-8385 (SK-N-AS and SK-N-BE(2) 4 μM; SH-EP and WAC2 12 μM) for 24 h. PP2A activation was significantly increased in SK-N-AS with ATUX-3364 (123.5 ± 0.7% vs. 100% ± 0%, ATUX-3364 vs. control, *p* ≤ 0.01) or ATUX-8385 (134.1 ± 5.5% vs. 100% ± 0%, ATUX-8385 vs. control, *p* ≤ 0.001) (Figure 2A) treatment. Similarly, PP2A was significantly activated in the three other cell lines: SK-N-BE(2) (135.4 ± 4.2% vs. 100% ± 0%, ATUX-3364 vs. control, *p* ≤ 0.05; 167.2 ± 13.7% vs. 100% ± 0%, ATUX-8385 vs. control, *p* ≤ 0.01, Figure 2B), SH-EP (107.4 ± 1% vs. 100% ± 0%, ATUX-3364 vs. control *p* ≤ 0.01; 126.6 ± 6.4% vs. 100% ± 0%, ATUX-8385 vs. control, *p* ≤ 0.05, Figure 2C), and WAC2 (140 ± 5.4% vs. 100% ± 0%, ATUX-3364 vs. control, *p* ≤ 0.001; 118.2 ± 9.2% vs. 100% ± 0%, ATUX-8385 vs. control, *p* ≤ 0.05, Figure 2D). There was no significant difference in PP2A activation when comparing the two compounds in any of the cell lines (Figure 2A–D). There was no significant difference in baseline PP2A activation between the established cell lines (Supporting Data, Figure S1).

### 3.3. Effects of ATUX-3364 and ATUX-8385 on PP2A Endogenous Inhibitors

Since we have previously seen changes in the endogenous inhibitors of PP2A, SET, and CIP2A, following PP2A activation [10,14], we used immunoblotting to examine the effects of the PP2A-activating compounds on these endogenous inhibitors. The results were variable and cell line dependent. CIP2A decreased following treatment with ATUX-3364 or ATUX-8385 in SK-N-AS (Figure 2B), SK-N-BE(2) (Figure 2C, left panel), and SH-EP (Figure 2D) cells. CIP2A decreased in WAC2 cells only with ATUX-8385 (Figure 2E). Similarly, CIP2A expression only decreases in COA6 (Figure 2F) cells with the highest concentration of ATUX-8385 (Figure 2F). Treatment with either compound had variable effects on SET (Figure 2B–F).

### 3.4. Treatment with ATUX-3364 or ATUX-8385 Decreases Cell Viability and Proliferation and Causes Apoptosis

**Figure 3:**
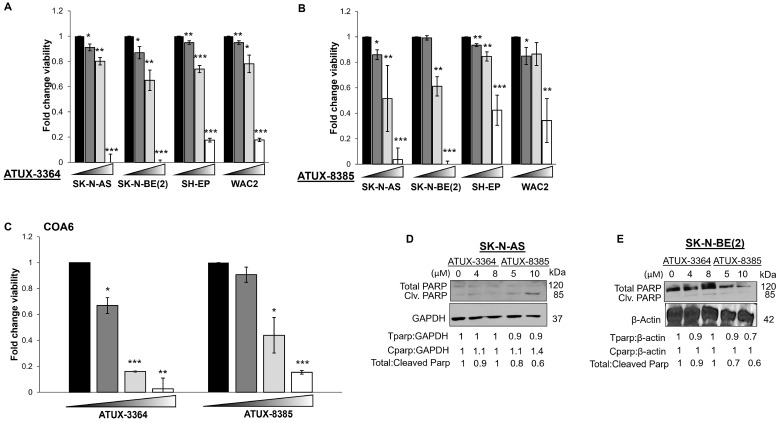
ATUX-3364 and ATUX-8385 decreased NB viability. Cells from established NB cell lines SK-N-AS, SK-N-BE(2), SH-EP, and WAC2 were treated with increasing doses of (**A**) ATUX-3364 (0–20 µM) or (**B**) ATUX-8385 (0–20 µM) for 24 h. Both compounds significantly decreased viability in all cell lines. (**C**) COA6 human NB PDX cells were treated with increasing doses of ATUX-3364 or ATUX-8385 (0–25 µM) for 24 h. Both compounds decreased viability. Immunoblotting was performed on whole cell lysates from (**D**) SK-N-AS and (**E**) SK-N-BE(2) NB cells following treatment with ATUX-3364 or ATUX-8385. Cleaved PARP (85 kDa) was increased, and total PARP (120 kDa) decreased in SK-N-AS and SK-N-BE(2) whole cell lysates. Data are reported as mean fold change ± SEM, and experiments were repeated with at least three biological replicates. * *p* ≤ 0.05, ** *p* ≤ 0.01, *** *p* ≤ 0.001. The uncropped bolts are shown in Supplementary Materials.

To evaluate the effects of these compounds on viability, the four NB cell lines and PDX cells were treated with increasing concentrations of ATUX-3364 or ATUX-8385 for 24 h. Treatment with ATUX-3364 (0, 5, 7.5, 20 μM) resulted in significantly decreased viability in all cell lines (Figure 3A). Similarly, ATUX-8385 significantly decreased viability at the same doses (Figure 3B). In the PDX COA6 cells, viability was significantly decreased following treatment with either compound (0, 5, 10, 20 μM) (Figure 3C). We examined whether apoptosis contributed to cell death. Apoptosis is characterized by a decrease in total PARP or an increase in cleaved PARP. There was an increase in cleaved PARP and a decrease in total PARP in SK-N-AS and SK-N-BE(2) cells after ATUX-8385 treatment, indicating apoptosis (Figure 3D,E). In addition, we investigated proliferation with CellTiter 96 following treatment. Since this assay employs the same chemical reaction as the alamarBlue assay, these data are provided as supplemental information. There was a significant decrease in proliferation in the four human NB cell lines and the PDX cells following treatment with either ATUX-3364 or ATUX-8385 (Supporting Data, Figure S2A–C).

### 3.5. Treatment with ATUX-3364 or ATUX-8385 Decreases Motility

**Figure 4:**
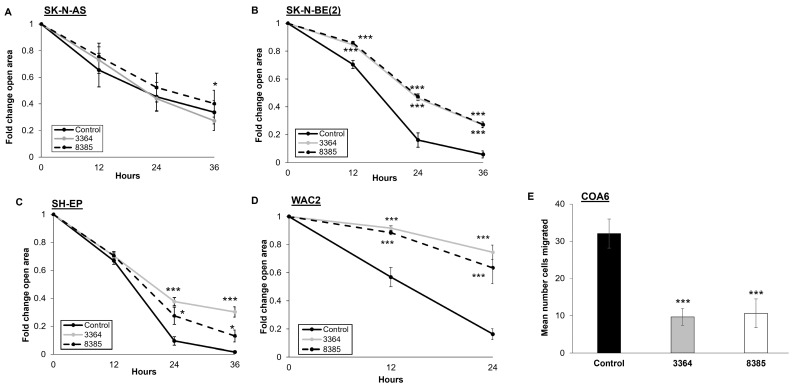
ATUX-3364 and ATUX-8385 decreased NB motility. Migration was examined using monolayer wound healing (scratch) assay for established NB cell lines. After a sterile scratch, plates were treated with ATUX-3364 or ATUX-8385, and micrographs of the plates were obtained. For quantification of open wound area, ImageJ MRI Wound Healing Tool (https://imagej.net, accessed on 26 November 2021) was used. (**A**) Migration of SK-N-AS cells was only affected by ATUX-8385 (4 μM). (**B**) Treatment of SK-N-BE(2) cells with ATUX-3364 or ATUX-8385 (4 μM) significantly decreased migration. (**C**) SH-EP and (**D**) WAC2 cells were treated with ATUX-3364 or ATUX-8385 (12 μM). Migration in both cell lines was significantly decreased following treatment. (**E**) Migration was assessed in COA6 PDX cells using modified Boyden chamber assays. Treatment with ATUX-3364 and ATUX-8385 (6 μM) significantly decreased COA6 migration. Data are reported as mean fold change in area of open area or mean number of cells migrated ± SEM, and experiments were repeated with at least three biological replicates. * *p* ≤ 0.05, *** *p* ≤ 0.001.

Wound healing assays were utilized to assess motility in the established NB cell lines. Motility in SK-N-AS cells was only affected in a statistically significant manner by ATUX-8385 (Figure 4A). Treatment of SK-N-BE(2), SH-EP, and WAC2 cells with ATUX-3364 or ATUX-8385 resulted in a significant decrease in motility compared to untreated cells (*p* ≤ 0.001, Figure 4B–D). Due to the non-adherent nature of COA6 cells, modified Boyden chamber assays were utilized to assess motility. COA6 cell migration was significantly decreased following treatment with ATUX-3364 or ATUX-8385 (6 μM) (*p* ≤ 0.001, Figure 4E).

### 3.6. In Vivo Studies and MYCN Protein Expression at the Molecular Level

**Figure 5:**
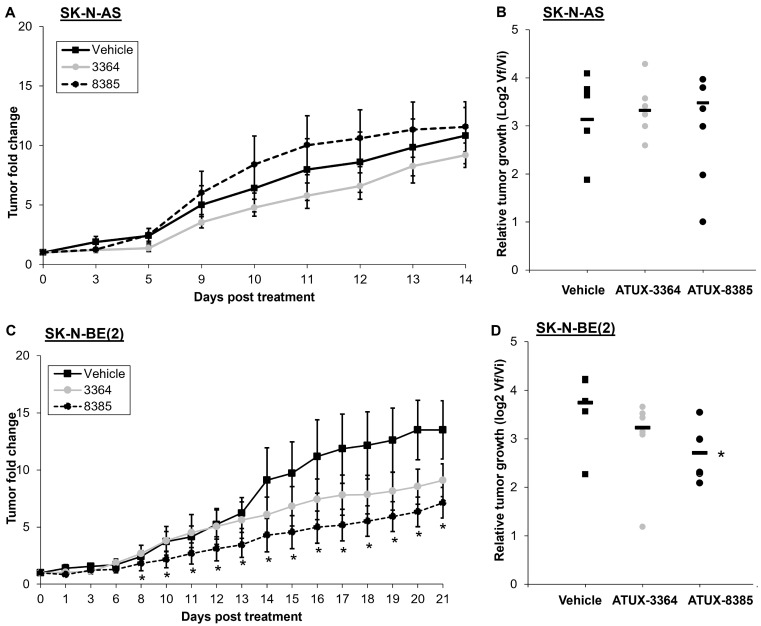
ATUX-8385 decreased SK-N-BE(2) tumor growth *in vivo*. SK-N-AS or SK-N-BE(2) cells were injected into the right flank of female athymic nude mice. When tumor volume reached 100 mm^3^, animals were randomized into groups of three to receive either vehicle, ATUX-3364, or ATUX-8385 (50 mg/kg). (**A**,**B**) Animals bearing SK-N-AS tumors (n = 7 in vehicle, n = 6 in ATUX-3364, n = 6 in ATUX-8385 treatment groups) were treated for 14 days. There was no significant difference in change in tumor volume or relative tumor growth with either compound. (**C**,**D**) Animals bearing SK-N-BE(2) tumors (n = 5 in vehicle, n = 6 in ATUX-3364, and n = 7 in ATUX-8385 treatment groups) were treated for 21 days. There was a significant decrease in tumor volume and relative tumor growth with ATUX-8385 treatment. Data are reported as mean fold change in tumor volume ± SEM. * *p* ≤ 0.05.

Because the *in vitro* findings demonstrated decreased survival and motility, we proceeded to *in vivo* studies. SK-N-AS or SK-N-BE(2) cells were injected into the flank of athymic nude mice. When tumors reached a volume of 100 mm^3^, animals were randomized to treatment with PP2A activators of 50 mg/kg oral dosing twice daily, which was based on dosages established in previous in vivo studies [10,21] and pharmacokinetic data (Figure 1). There was no decrease in tumor volumes (Figure 5A) or relative tumor growth (Figure 5B) in animals bearing SK-N-AS tumors following 14 days of treatment with either compound. In animals bearing SK-N-BE(2) tumors, ATUX-8385 treatment led to a significant decrease in tumor volumes (Figure 5C) as well as relative tumor growth (Figure 5D), while ATUX-3364 trended toward a decrease but did not reach statistical significance (Figure 5C,D).

**Figure 6:**
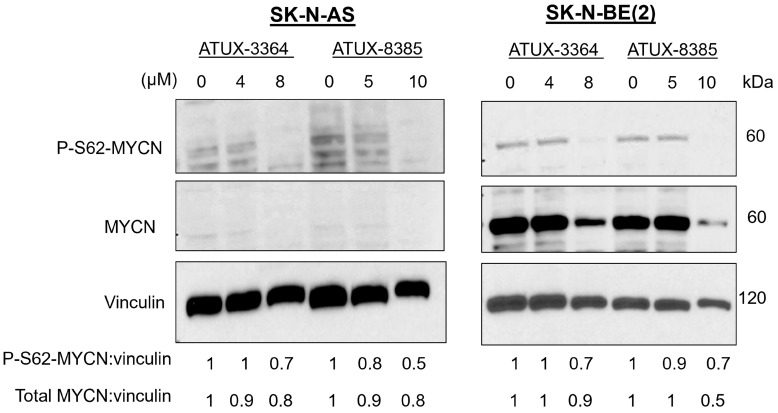
ATUX-3364 and ATUX-8385 decreased serine phosphorylation of MYCN. SK-N-AS (*MYCN*-non-amplified) and SK-N-BE(2) (*MYCN*-amplified) cells were treated with increasing concentrations of ATUX-3364 or ATUX-8385. Whole cell lysates were prepared, and immunoblotting completed for phosphorylated MYCN (P-S62) and total MYCN protein expression. Vinculin was assayed as a loading control. Serine 62 phosphorylation of MYCN was decreased with treatment with both drugs. Total MYCN protein expression was also decreased following treatment. The uncropped bolts are shown in Supplementary Materials.

Because of the more pronounced response in the *MYCN-amplified* (SK-N-BE(2)) compared to the *MYCN*-non-amplified (SK-N-AS) tumors in vivo, we postulated that PP2A-activating compounds may be affecting MYCN. We treated SK-N-AS *MYCN*-non-amplified and SK-N-BE(2) *MYCN-amplified* cells with ATUX-3364 or ATUX-8385 and analyzed MYCN phosphorylation with immunoblotting. Upon treatment, both phosphorylated MYCN (S62) and total MYCN protein expression decreased in both cell lines (Figure 6. Dephosphorylation of MYCN at S62 results in the destabilization of the protein leading to degradation [6]. This degradation is likely responsible for the decrease in total MYCN protein.

## 4. Discussion

The results from the present study are critically important to the development of improved activators of PP2A. The compounds reported here, ATUX-8385 and ATUX-3364, are novel tricyclic sulfonamides derived from a medicinal chemistry program to reverse engineer tricyclic neuroleptics as anti-cancer agents [13,17]. These compounds bind PP2A AC heterodimers, activating PP2A [19,20] via stabilization of PP2A ABC holoenzyme heterotrimers [19,20]. We previously reported decreased NB tumorigenesis with PP2A activation [9,10]. The current compounds are novel compared to their predecessors in relation to their ability to be formulated as a salt which has implications for clinical administration. Additionally, they are significantly more stable than the earlier sulfonamide compounds. Finally, they lack the effects on the immune system seen with earlier PP2A activators such as FTY-720.

PP2A reactivation has emerged as a treatment strategy for various cancers. The functional inhibition of PP2A and its tumor suppressor activity is mainly by non-genomic mechanisms [27] and is evident in a diverse range of cancers [17,28,29,30,31]. One mechanism for this loss of function is alteration of endogenous PP2A inhibitors, including CIP2A and SET. CIP2A overexpression is associated with poor patient outcomes in numerous solid tumors, including NB [9], hepatocellular carcinoma [32], and lung cancer [33]. Similarly, overexpression of SET has been linked to increased cell proliferation and tumorigenesis [34,35]. There is evidence that the knockdown of these inhibitors restored the tumor suppressor function of PP2A, and NB cells treated with SET or CIP2A siRNA had a decreased malignant phenotype [9]. Our previous studies showed that certain PP2A activators functioned to decrease the expression of these endogenous inhibitors in NB [10] as well as hepatoblastoma [14]. In the current study, the PP2A-activating compounds studied were not associated with a consistent change in SET protein expression. For instance, PP2A activators decreased SET expression in the MYCN-overexpressed WAC2 cells but were unchanged in the isogenic *MYCN*-non-amplified SH-EP cells. This finding argues against a MYCN status dependence and gets support from our earlier findings wherein it did not appear as though SET expression was MYCN dependent [9]. In contrast, findings in the current study on the effects on the other endogenous PP2A inhibitor, CIP2A, were different, in that ATUX-3364 or ATUX-8385 treatment decreased CIP2A expression in the majority of the cell lines tested. One explanation for this finding is that normally, CIP2A displaces the PP2A-A subunit and thereby hijacks both the PP2A-B56α and the catalytic PP2Ac subunit to form a CIP2A-B56α-PP2Ac pseudotrimer that stabilizes CIP2A and promotes its function [36]. These ATUX compounds bind the PP2A-B56a subunit, thereby disrupting the CIP2A heterodimer and functioning to destabilize CIP2A. The current data, together with previous findings, suggest novel therapeutics that function to antagonize CIP2A, with consequent restoration of PP2A activity, hold potential for translation into the clinic.

Our previous investigation using an earlier non-basic PP2A-activating compound, DBK-1154, showed decreased MYCN expression in NB [10]. The same study demonstrated that this activator was better at decreasing tumor growth in animals bearing *MYCN*-amplified tumors than non-amplified tumors. These findings were attributed to the dependence of amplified cells on MYCN [10]. In the current investigations employing the newer tricyclic sulfonamides, ATUX-3364 and ATUX-8385, tumor volumes, as well as relative tumor growth in animals bearing *MYCN*-amplified tumors, were significantly decreased. The same results were not observed in the animals bearing non-amplified tumors. On a molecular level, PP2A-based reactivation led to dephosphorylation of MycN-S62. Dephosphorylation of MYCN at serine 62 results in the eventual targeting of MYCN for degradation [37]. This mechanism may serve to target MYCN-addicted NB, as *MYCN*-amplified NB cells rely on a discrete set of genes that are either unique to or are only minimally shared with other tumor types. Hence, our future investigations will involve identifying the effects of PP2A activation on genes responsible for the diverse vulnerabilities in *MYCN*-amplified NB, specific for those that contribute to the initiation as well as maintenance of the transformed phenotype in *MYCN-amplified* NB.

*MYCN* amplification is one of the most common events associated with NB tumorigenesis, providing many reasons to focus on tumor-specific MYCN dependency and to target this entity as an effective treatment for NB. However, historically, MYCN protein has been considered a non-druggable target [38]. Hence, therapeutic approaches for targeting MYCN-driven tumors have focused on the disruption of transcription, translation, or protein stability. The present work employing a panel of NB cell lines with varying *MYCN* amplification along with a *MYCN*-amplified human NB PDX demonstrates that *MYCN* may be targeted to gain therapeutic efficacy. We demonstrate that the MYCN protein degradation using novel small molecule activators of PP2A has significant single-agent preclinical activity. Moreover, this approach comes with added advantages such as (1) PP2A activators are orally bioavailable and well tolerated across preclinical models of pediatric cancer, including NB [9,30]; (2) PP2A reactivation targets MYCN at the protein level, resulting in its degradation; and (3) the specificity of these small molecules to PP2A has been validated across pediatric cancers, again including NB [9,30]. Together, the novel PP2A activators and pharmaceutically druggable approach in the current study target MYCN degradation, leading to a significant reduction in overall cell viability, proliferation, and migratory potential and a reduction in tumor volume in vivo.

High MycN/CIP2A levels correlate with poor NB prognosis [39]. Kerosuo and colleagues demonstrated an interaction between these two proteins and highlighted that CIP2A knockdown affected MYCN expression. These findings may add to the explanation for decreased total MYCN expression after treatment with ATUX-3362 and ATUX-8385. To emphasize the clinical relevance of our findings, we examined a potential correlation between the expression of CIP2A and relapse-free survival (RFS) in NB patient samples using the publicly available dataset Versteeg88. We noted that patients with low *CIP2A* (KIAA1524) expression had higher RFS probability (Supporting Data, Figure S3) [40]. Of note, in the current studies, expression of both CIP2A and MYCN was found to be least affected in the SK-N-AS cell line (*MYCN* non-amplified) upon PP2A activation with ATUX-8385, fostering a correlation between CIP2A and MYCN, paving the way for promising future investigations.

## 5. Conclusions

We have shown that two novel PP2A-activating compounds affect the viability, proliferation, and motility of several established *MYCN-amplified* and non-amplified NB cell lines and a *MYCN-amplified* human NB PDX line. In the *MYCN*-driven NB model employed, MYCN expression is inhibited by pharmacologic PP2A activation, likely contributing to the phenotypic findings. In vivo experiments highlight that the consequences of *MYCN* oncogene inactivation are more dramatic in oncogene-addicted tumors, as observed in *MYCN*-amplified SK-N-BE(2) tumor-bearing mice. These mechanistic insights may be useful for developing new PP2A-based therapies that may target MYCN.

## Data Availability

There were no data sets generated for the current studies.

## Supplementary Materials

The following supporting information can be downloaded at: https://www.mdpi.com/article/10.3390/cancers16223836/s1, Figure S1: Baseline PP2A activity; Figure S2: ATUX-3364 and ATUX-8385 led to decreased NB proliferation; Figure S3: CIP2A (KIAA1524) expression correlates with relapse free survival.

## References

[1] Cohn S.L., Pearson A.D.J., London W.B., Monclair T., Ambros P.F., Brodeur G.M., Faldum A., Hero B., Iehara T., Machin D. (2009). The International Neuroblastoma Risk Group (INRG) Classification System: An INRG Task Force Report. J. Clin. Oncol..

[2] Smith V., Foster J. (2018). High-Risk Neuroblastoma Treatment Review. Children.

[3] Colon N.C., Chung D.H. (2011). Neuroblastoma. Adv. Pediatr..

[4] Matthay K.K., Maris J.M., Schleiermacher G., Nakagawara A., Mackall C.L., Diller L., Weiss W.A. (2016). Neuroblastoma. Nat. Rev. Dis. Primers.

[5] Weiss W.A. (1997). Targeted Expression of MYCN Causes Neuroblastoma in Transgenic Mice. EMBO J..

[6] Gustafson W.C., Weiss W.A. (2010). Myc Proteins as Therapeutic Targets. Oncogene.

[7] Li M., Makkinje A., Damuni Z. (1996). The Myeloid Leukemia-Associated Protein SET Is a Potent Inhibitor of Protein Phosphatase 2A. J. Biol. Chem..

[8] Kauko O., O’Connor C.M., Kulesskiy E., Sangodkar J., Aakula A., Izadmehr S., Yetukuri L., Yadav B., Padzik A., Laajala T.D. (2018). PP2A Inhibition Is a Druggable MEK Inhibitor Resistance Mechanism in KRAS-Mutant Lung Cancer Cells. Sci. Transl. Med..

[9] Williams A.P., Garner E.F., Waters A.M., Stafman L.L., Aye J.M., Markert H., Stewart J.E., Beierle E.A. (2019). Investigation of PP2A and Its Endogenous Inhibitors in Neuroblastoma Cell Survival and Tumor Growth. Transl. Oncol..

[10] Bownes L.V., Marayati R., Quinn C.H., Beierle A.M., Hutchins S.C., Julson J.R., Erwin M.H., Stewart J.E., Mroczek-Musulman E., Ohlmeyer M. (2022). Pre-Clinical Study Evaluating Novel Protein Phosphatase 2A Activators as Therapeutics for Neuroblastoma. Cancers.

[11] Pippa R., Odero M.D. (2020). The Role of MYC and PP2A in the Initiation and Progression of Myeloid Leukemias. Cells.

[12] Chien W., Sun Q.-Y., Lee K.L., Ding L.-W., Wuensche P., Torres-Fernandez L.A., Tan S.Z., Tokatly I., Zaiden N., Poellinger L. (2015). Activation of Protein Phosphatase 2A Tumor Suppressor as Potential Treatment of Pancreatic Cancer. Mol. Oncol..

[13] Sangodkar J., Perl A., Tohme R., Kiselar J., Kastrinsky D.B., Zaware N., Izadmehr S., Mazhar S., Wiredja D.D., O’Connor C.M. (2017). Activation of Tumor Suppressor Protein PP2A Inhibits KRAS-Driven Tumor Growth. J. Clin. Investig..

[14] Stafman L.L., Williams A.P., Marayati R., Aye J.M., Stewart J.E., Mroczek-Musulman E., Beierle E.A. (2019). PP2A Activation Alone and in Combination with Cisplatin Decreases Cell Growth and Tumor Formation in Human HuH6 Hepatoblastoma Cells. PLoS ONE.

[15] Tohmé R., Izadmehr S., Gandhe S., Tabaro G., Vallabhaneni S., Thomas A., Vasireddi N., Dhawan N.S., Ma’ayan A., Sharma N. (2019). Direct Activation of PP2A for the Treatment of Tyrosine Kinase Inhibitor–Resistant Lung Adenocarcinoma. JCI Insight.

[16] Garner E.F., Williams A.P., Stafman L.L., Aye J.M., Mroczek-Musulman E., Moore B.P., Stewart J.E., Friedman G.K., Beierle E.A. (2018). FTY720 Decreases Tumorigenesis in Group 3 Medulloblastoma Patient-Derived Xenografts. Sci. Rep..

[17] Kastrinsky D.B., Sangodkar J., Zaware N., Izadmehr S., Dhawan N.S., Narla G., Ohlmeyer M. (2015). Reengineered Tricyclic Anti-Cancer Agents. Bioorg. Med. Chem..

[18] McClinch K., Avelar R.A., Callejas D., Izadmehr S., Wiredja D., Perl A., Sangodkar J., Kastrinsky D.B., Schlatzer D., Cooper M. (2018). Small-Molecule Activators of Protein Phosphatase 2A for the Treatment of Castration-Resistant Prostate Cancer. Cancer Res..

[19] Allen-Petersen B.L., Risom T., Feng Z., Wang Z., Jenny Z.P., Thoma M.C., Pelz K.R., Morton J.P., Sansom O.J., Lopez C.D. (2019). Activation of PP2A and Inhibition of mTOR Synergistically Reduce MYC Signaling and Decrease Tumor Growth in Pancreatic Ductal Adenocarcinoma. Cancer Res..

[20] Risom T., Wang X., Liang J., Zhang X., Pelz C., Campbell L.G., Eng J., Chin K., Farrington C., Narla G. (2019). Deregulating MYC in a Model of HER2+ Breast Cancer Mimics Human Intertumoral Heterogeneity. J. Clin. Investig..

[21] Merisaari J., Denisova O.V., Doroszko M., Le Joncour V., Johansson P., Leenders W.P.J., Kastrinsky D.B., Zaware N., Narla G., Laakkonen P. (2020). Monotherapy Efficacy of Blood–Brain Barrier Permeable Small Molecule Reactivators of Protein Phosphatase 2A in Glioblastoma. Brain Commun..

[22] Schweigerer L., Breit S., Wenzel A., Tsunamoto K., Ludwig R., Schwab M. (1990). Augmented MYCN Expression Advances the Malignant Phenotype of Human Neuroblastoma Cells: Evidence for Induction of Autocrine Growth Factor Activity. Cancer Res..

[23] Stafman L.L., Williams A.P., Marayati R., Aye J.M., Markert H.R., Garner E.F., Quinn C.H., Lallani S.B., Stewart J.E., Yoon K.J. (2019). Focal Adhesion Kinase Inhibition Contributes to Tumor Cell Survival and Motility in Neuroblastoma Patient-Derived Xenografts. Sci. Rep..

[24] Bownes L.V., Williams A.P., Marayati R., Stafman L.L., Markert H., Quinn C.H., Wadhwani N., Aye J.M., Stewart J.E., Yoon K.J. (2021). EZH2 inhibition decreases neuroblastoma proliferation and in vivo tumor growth. PLoS ONE.

[25] Schneider C.A., Rasband W.S., Eliceiri K.W. (2012). NIH Image to ImageJ: 25 Years of Image Analysis. Nat. Methods.

[26] Tang L., Zhang H., Zhang B. (2019). A Note on Error Bars as a Graphical Representation of the Variability of Data in Biomedical Research: Choosing between Standard Deviation and Standard Error of the Mean. J. Pancreatol..

[27] Kauko O., Westermarck J. (2018). Non-Genomic Mechanisms of Protein Phosphatase 2A (PP2A) Regulation in Cancer. Int. J. Biochem. Cell Biol..

[28] Arroyo J.D., Hahn W.C. (2005). Involvement of PP2A in Viral and Cellular Transformation. Oncogene.

[29] Sents W., Meeusen B., Kalev P., Radaelli E., Sagaert X., Miermans E., Haesen D., Lambrecht C., Dewerchin M., Carmeliet P. (2017). PP2A Inactivation Mediated by *PPP2R4* Haploinsufficiency Promotes Cancer Development. Cancer Res..

[30] Perrotti D., Neviani P. (2013). Protein Phosphatase 2A: A Target for Anticancer Therapy. Lancet Oncol..

[31] Bownes L.V., Julson J.R., Quinn C.H., Hutchins S.C., Erwin M.H., Markert H.R., Stewart J.E., Mroczek-Musulman E., Aye J., Yoon K.J. (2023). The Effects of Protein Phosphatase 2A Activation with Novel Tricyclic Sulfonamides on Hepatoblastoma. J. Pediatr. Surg..

[32] He H., Wu G., Li W., Cao Y., Liu Y. (2012). CIP2A Is Highly Expressed in Hepatocellular Carcinoma and Predicts Poor Prognosis. Diagn. Mol. Pathol..

[33] Dong Q.-Z., Wang Y., Dong X.-J., Li Z.-X., Tang Z.-P., Cui Q.-Z., Wang E.-H. (2011). CIP2A Is Overexpressed in Non-Small Cell Lung Cancer and Correlates with Poor Prognosis. Ann. Surg. Oncol..

[34] Carlson S.G., Eng E., Kim E.G., Perlman E.J., Copeland T.D., Ballermann B.J. (1998). Expression of SET, an Inhibitor of Protein Phosphatase 2A, in Renal Development and Wilms’ Tumor. J. Am. Soc. Nephrol..

[35] Fukukawa C., Shima H., Tanuma N., Ogawa K., Kikuchi K. (2000). Up-Regulation of I-2PP2A/SET Gene Expression in Rat Primary Hepatomas and Regenerating Livers. Cancer Lett..

[36] Pavic K., Gupta N., Omella J.D., Derua R., Aakula A., Huhtaniemi R., Määttä J.A., Höfflin N., Okkeri J., Wang Z. (2023). Structural Mechanism for Inhibition of PP2A-B56α and Oncogenicity by CIP2A. Nat. Commun..

[37] Liu Z., Chen S.S., Clarke S., Veschi V., Thiele C.J. (2020). Targeting MYCN in Pediatric and Adult Cancers. Front. Oncol..

[38] Liu R., Shi P., Wang Z., Yuan C., Cui H. (2021). Molecular Mechanisms of MYCN Dysregulation in Cancers. Front. Oncol..

[39] Kerosuo L., Neppala P., Hsin J., Mohlin S., Vieceli F.M., Török Z., Laine A., Westermarck J., Bronner M.E. (2018). Enhanced Expression of MycN/CIP2A Drives Neural Crest toward a Neural Stem Cell-like Fate: Implications for Priming of Neuroblastoma. Proc. Natl. Acad. Sci. USA.

[40] Molenaar J.J., Koster J., Zwijnenburg D.A., van Sluis P., Valentijn L.J., van der Ploeg I., Hamdi M., van Nes J., Westerman B.A., van Arkel J. (2012). Sequencing of Neuroblastoma Identifies Chromothripsis and Defects in Neuritogenesis Genes. Nature.

