# Supplementary material for: Novel PP2A-Activating Compounds in Neuroblastoma
Source: Cancers (Basel). 2024 Nov 15;16(22):3836. doi: 10.3390/cancers16223836 (PMC11592631; doi:10.3390/cancers16223836)
Supplement: Supplementary file 1 [file cancers-16-03836-s001.zip › cancers-3239042-original-images.pdf]

Figure 2

B

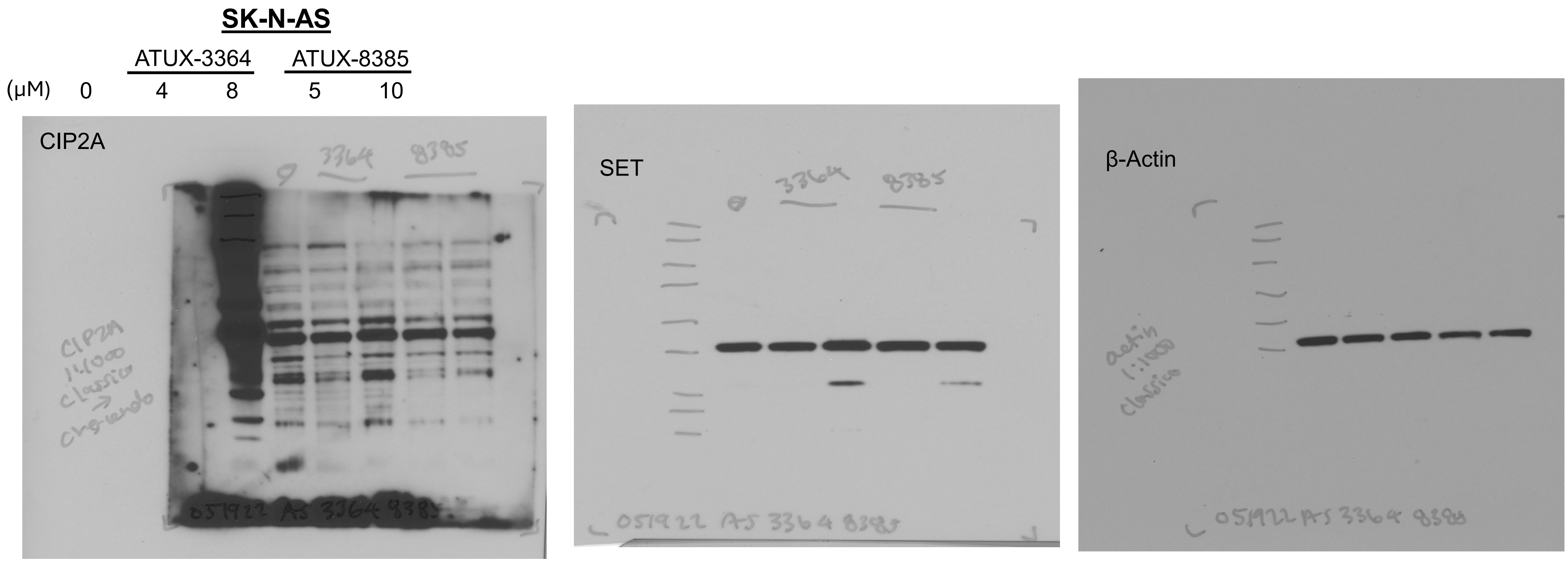

Figure 2

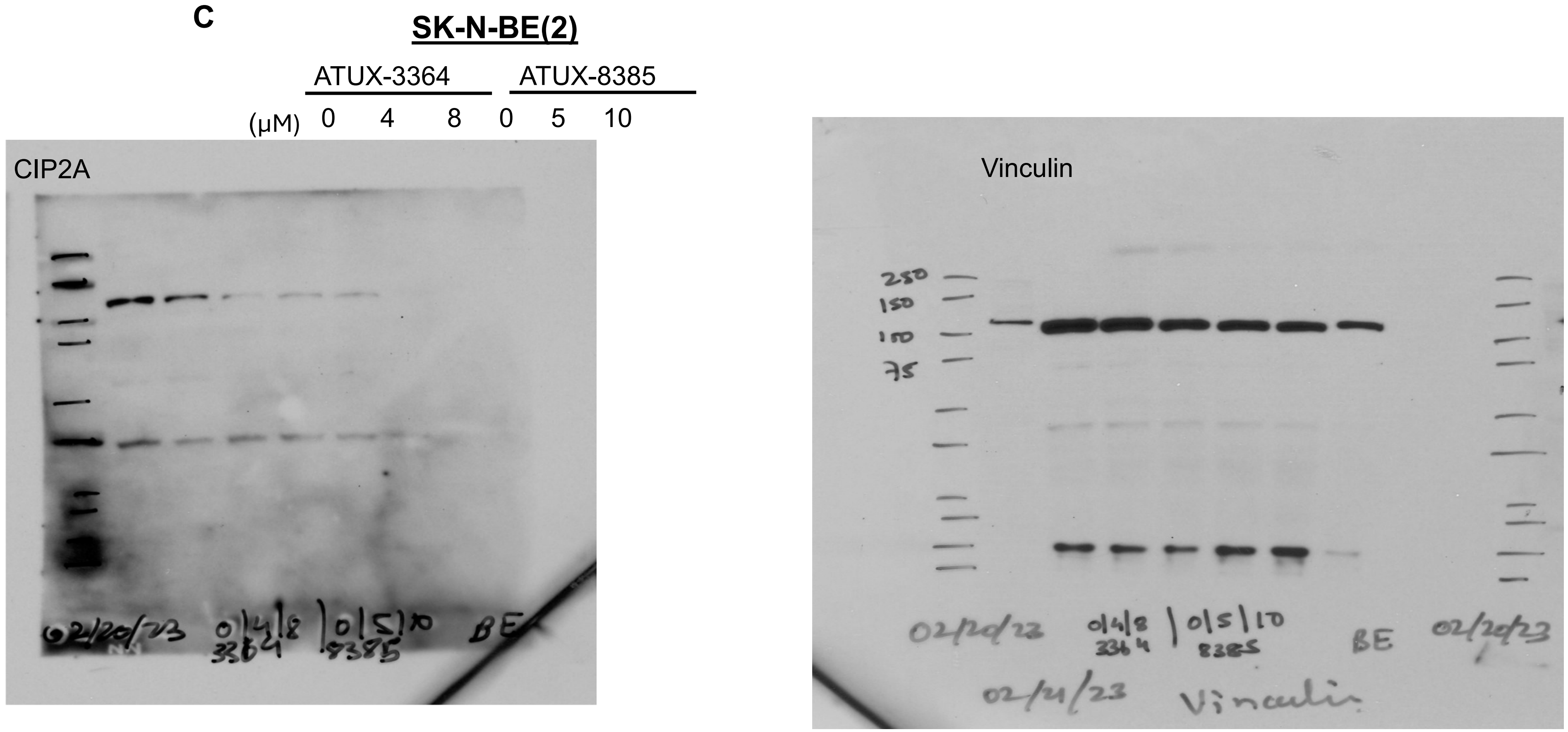

Figure 2

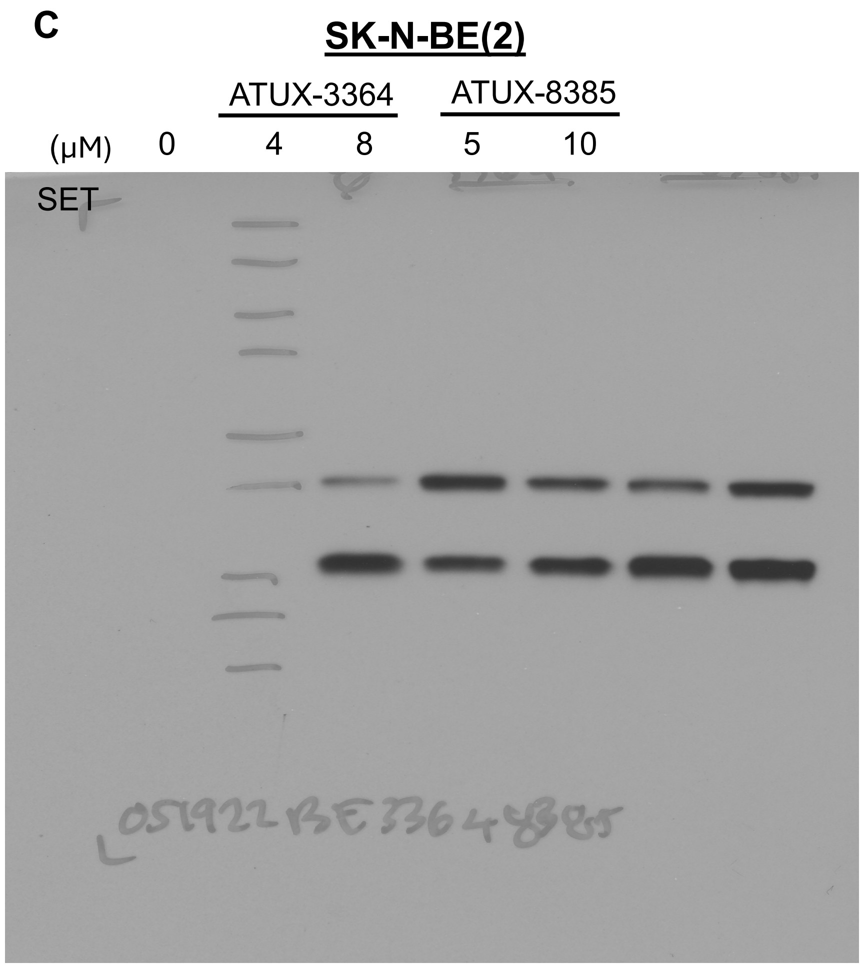
C
SK-N-BE(2)
ATUX-3364
ATUX-8385
(µM) 0 4 8 5 10
SET
051922 BE 3364 8385

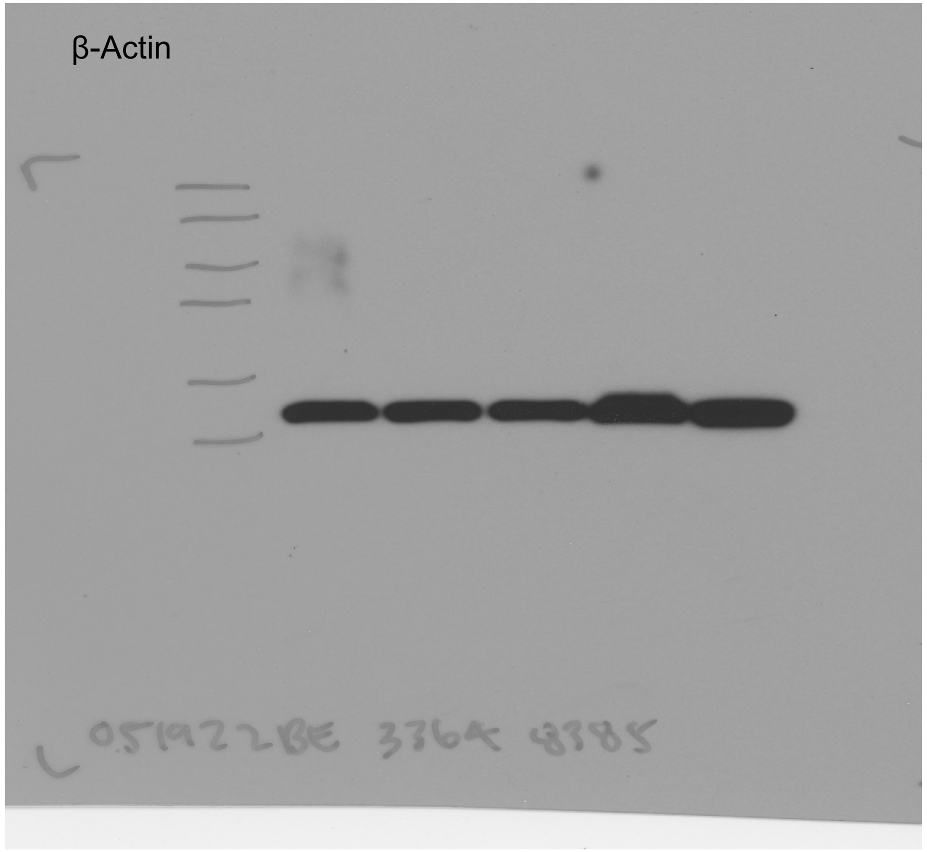
β-Actin
051922BE 3364 8385

Figure 2

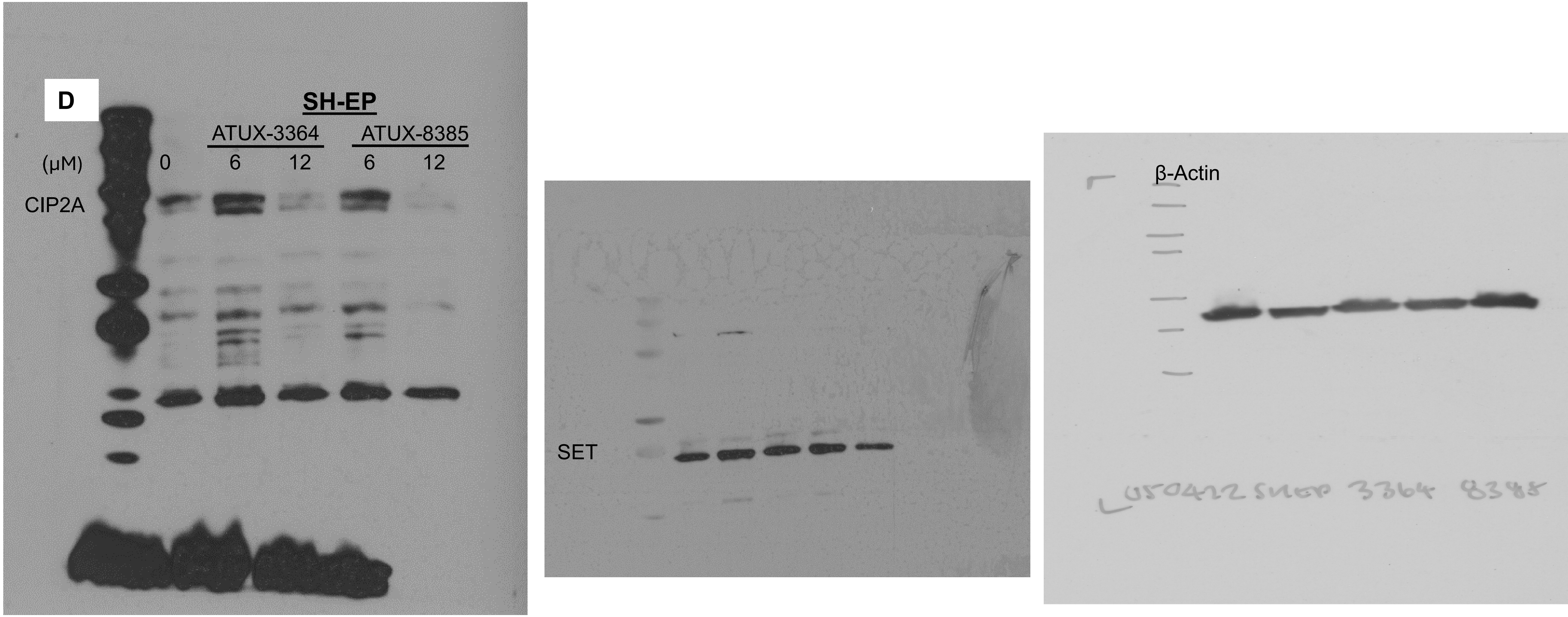

Figure 2

E

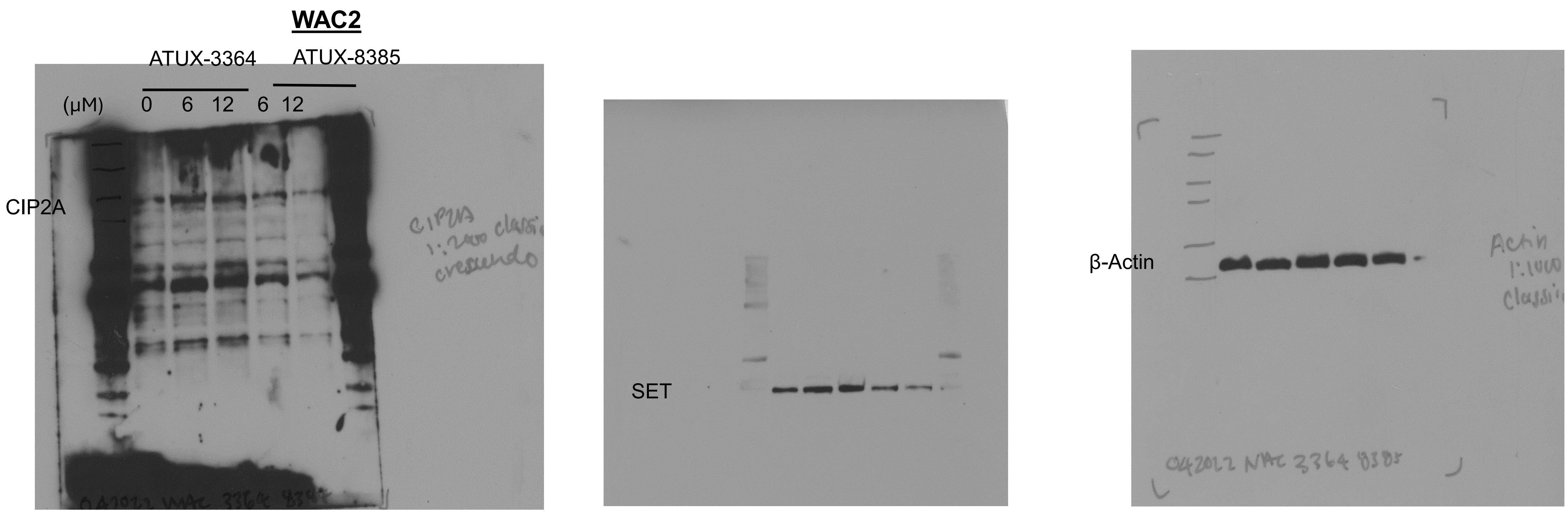

Figure 2

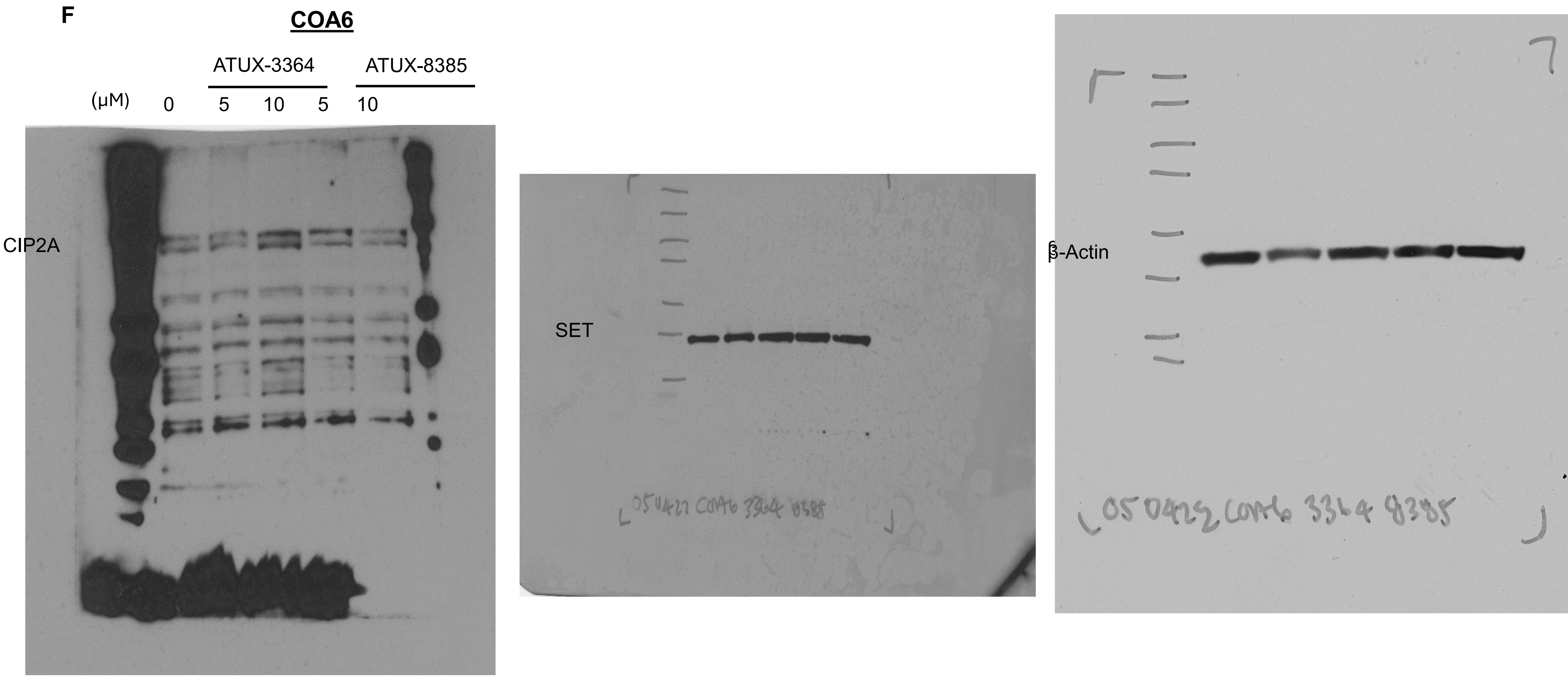

Figure 3

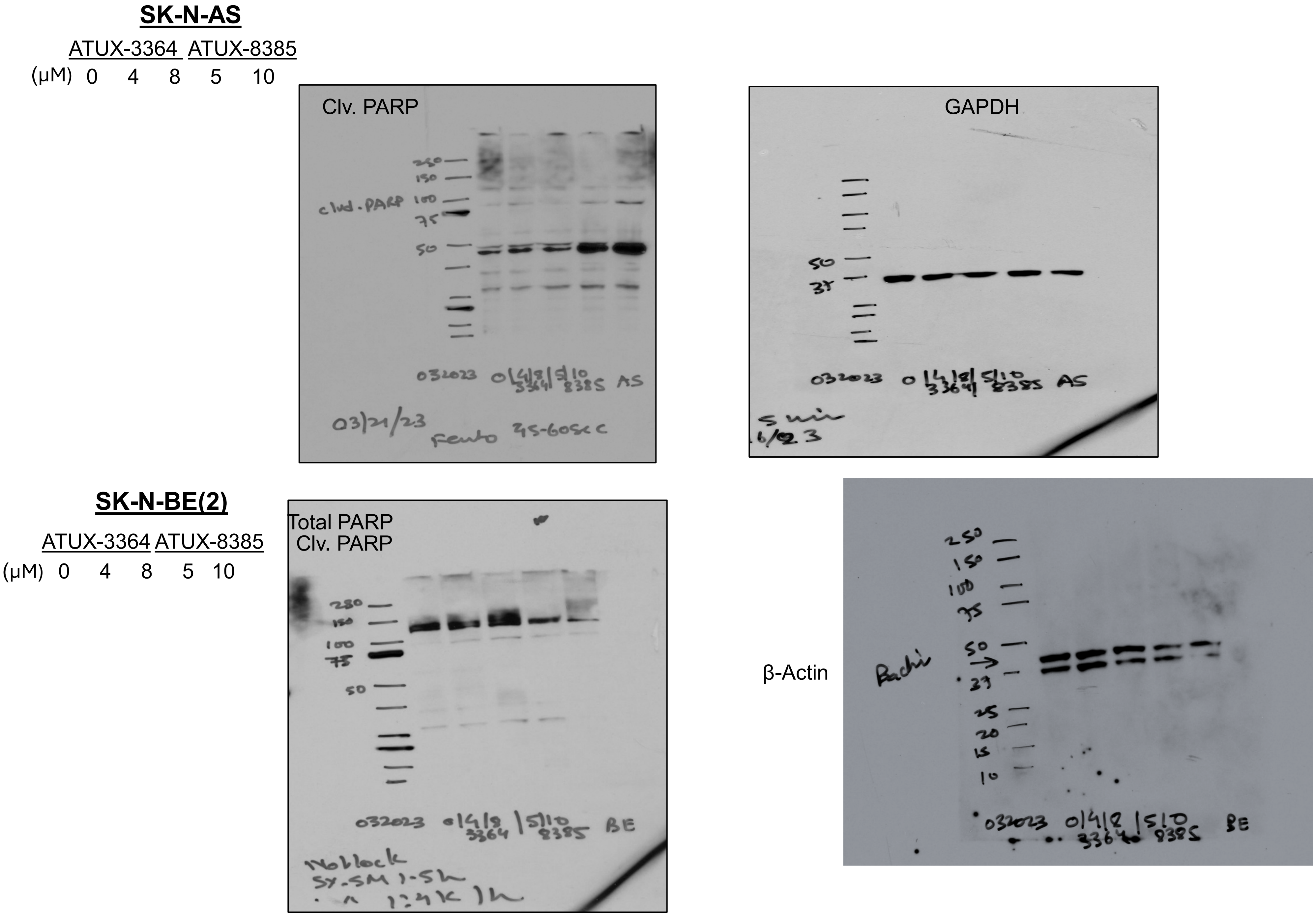
SK-N-AS
ATUX-3364 ATUX-8385
(µM) 0 4 8 5 10
Clv. PARP
GAPDH
SK-N-BE(2)
ATUX-3364 ATUX-8385
(µM) 0 4 8 5 10
Total PARP
Clv. PARP
β-Actin

Figure 6

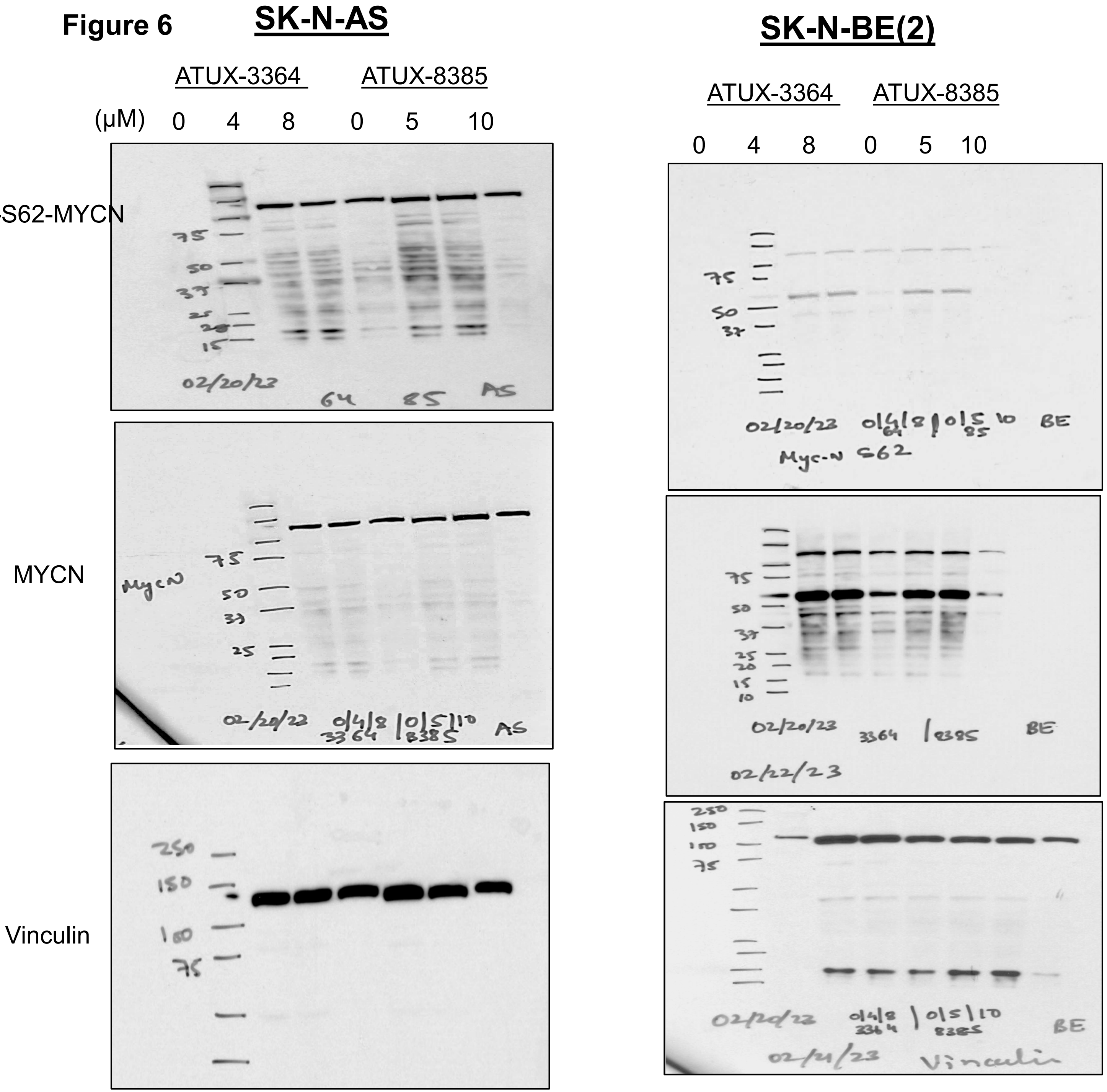

SK-N-AS
ATUX-3364
ATUX-8385
(µM) 0 4 8 0 5 10
S62-MYCN
MYCN
Vinculin
SK-N-BE(2)
ATUX-3364
ATUX-8385
0 4 8 0 5 10
