# Supplementary material for: Novel PP2A-Activating Compounds in Neuroblastoma
Source: Cancers (Basel). 2024 Nov 15;16(22):3836. doi: 10.3390/cancers16223836 (PMC11592631; doi:10.3390/cancers16223836)
Supplement: Supplementary file 1 [file cancers-16-03836-s001.zip › Supporting Data.pdf]

Supporting Data

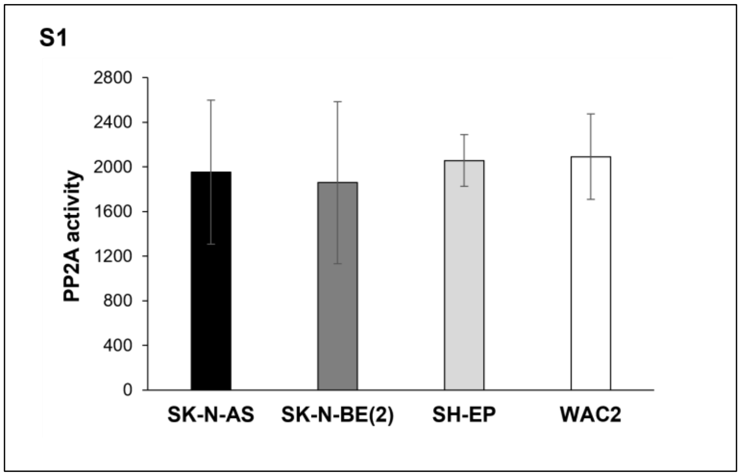

Supporting Data Figure: S1. Baseline PP2A activity. SK-N-AS, SK-N-BE(2), SH-EP, or WAC2 cells (1 × 10^6) were plated for 24 hours and PP2A activity measured. There was no statistically significant difference between PP2A activity at baseline between the cell lines.

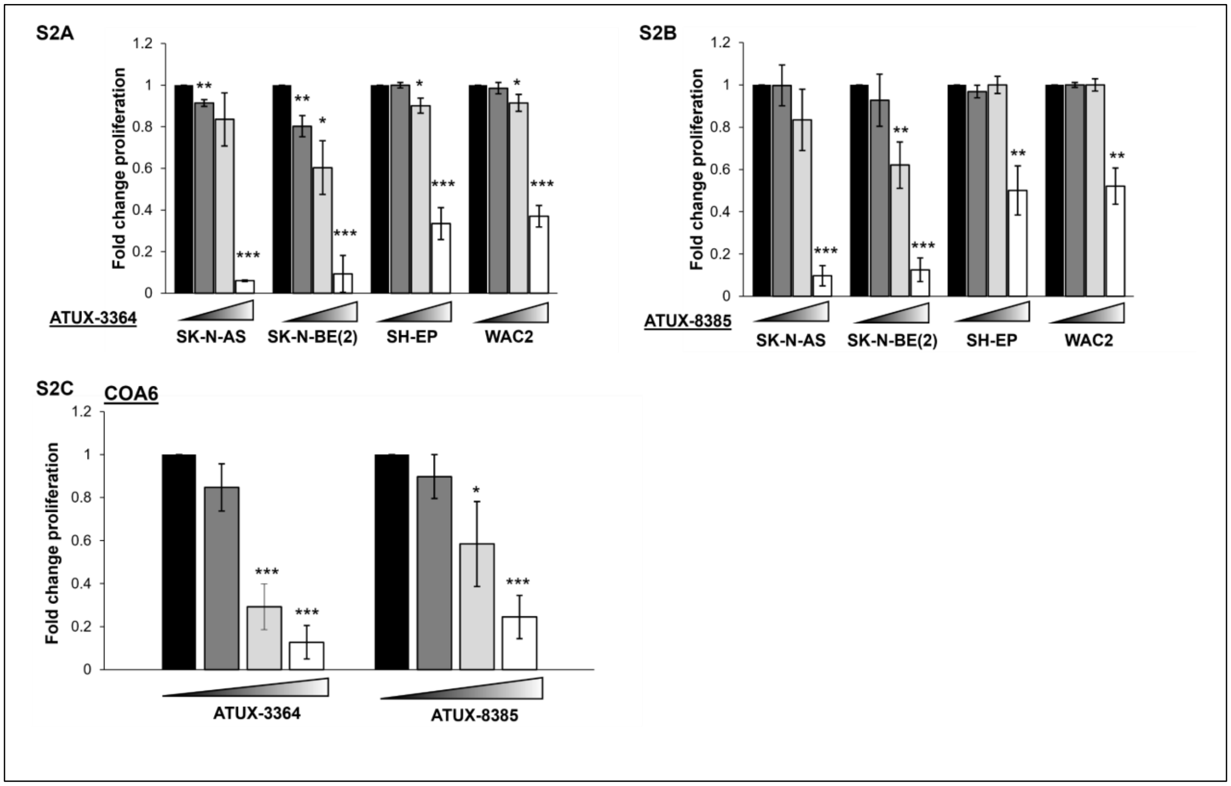

Supporting Data Figure: S2. ATUX-3364 and ATUX-8385 led to decreased NB proliferation. Cells from established NB cell lines SK-N-AS, SK-N-BE(2), SH-EP and WAC2 (5×10^3 cells) were plated in 96-well plates and treated with increasing doses of ATUX-3364 (**S2A**) or ATUX-8385 (**S2B**) for 24 hours. Both compounds significantly decreased proliferation. (**S2C**) COA6 human NB PDX cells (1×10^4 cells) were plated in 96-well plates and treated with increasing doses of ATUX-3364 or ATUX-8385 for 24 hours. Both compounds decreased proliferation. Data are reported as mean fold change ± SEM, and experiments were repeated with at least three biologic replicates. *p≤0.05, **p≤0.01, *** p≤0.001

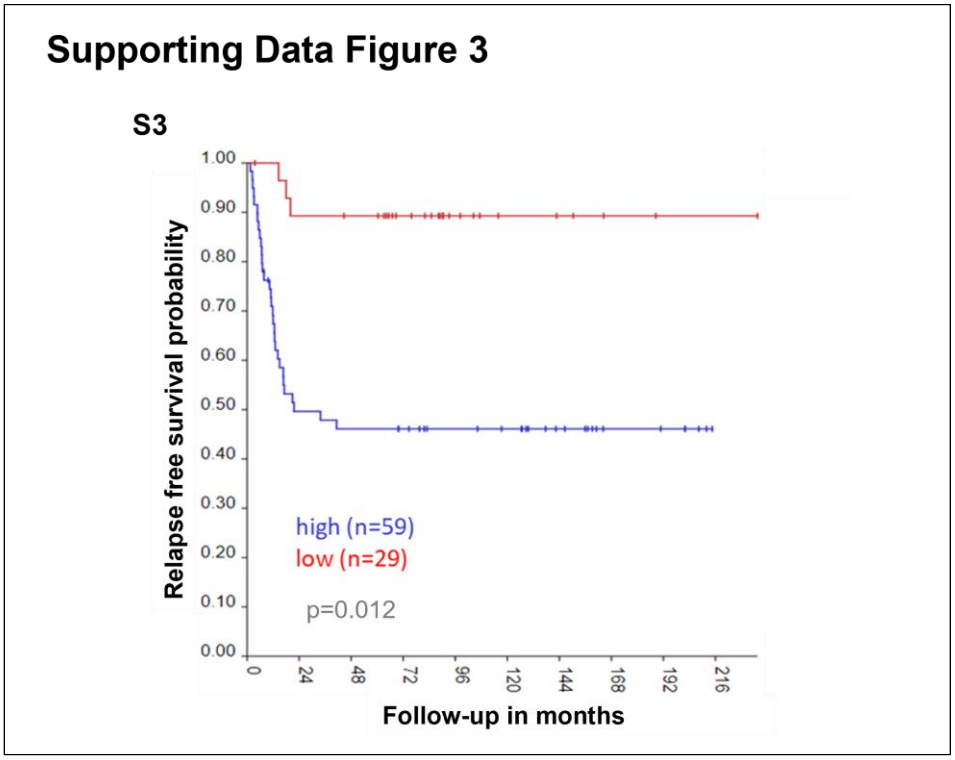

Supporting Data Figure: S3. CIP2A (*KIAA1524*) expression correlates with relapse free survival. Kaplan-Meier survival curve demonstrating a higher relapse free survival for patients with low CIP2A levels, where low expression is defined as greater than the median (n = 29; tumors) and high expression as less than the median (n=59; tumors) in the clinical dataset using the R2: microarray analysis and visualization platform (http://r2.amc.nl).
